# DDX3 regulates endoplasmic reticulum stress-induced ATF4 expression

**DOI:** 10.1038/s41598-017-14262-7

**Published:** 2017-10-23

**Authors:** Pauline Adjibade, Valérie Grenier St-Sauveur, Jonathan Bergeman, Marc-Etienne Huot, Edouard W. Khandjian, Rachid Mazroui

**Affiliations:** 1 Centre de recherche en cancérologie. Centre de recherche du CHU de Québec. Département de biologie moléculaire, biochimie médicale et pathologie, Faculté de médecine, Université Laval, Québec, PQ Canada; 2 Centre de Recherche, Institut universitaire en santé mentale de Québec. Département de psychiatrie et de neurosciences, Faculté de médecine, Université Laval, Québec, PQ Canada; 3 Present Address: Complexe de diagnostic et d’épidémiosurveillance vétérinaires du Québec (CDEVQ) Université de Montréal, Montréal, Canada

## Abstract

Accumulation of unfolded and potentially toxic proteins in the endoplasmic reticulum (ER) activates a cell stress adaptive response, which involves a reprogramming of general gene expression. ATF4 is a master stress-induced transcription factor that orchestrates gene expression in cells treated with various ER stress inducers including those used to treat cancers. ER stress-induced ATF4 expression occurs mainly at the translational level involving the activity of the phosphorylated (P) translation initiation factor (eIF) eIF2α. While it is well established that under ER stress PeIF2α drives ATF4 expression through a specialised mode of translation re-initiation, factors (e.g. RNA-binding proteins and specific eIFs) involved in PeIF2α-mediated ATF4 translation remain unknown. Here we identified the RNA-binding protein named DDX3 as a promotor of ATF4 expression in cancer cells treated with sorafenib, an ER stress inducer used as a chemotherapeutic. Depletion experiments showed that DDX3 is required for PeIF2α-mediated ATF4 expression. Luciferase and polyribosomes assays showed that DDX3 drives ER stress-induced ATF4 mRNA expression at the translational level. Protein-interaction assays showed that DDX3 binds the eIF4F complex, which we found to be required for ER stress-induced ATF4 expression. This study thus showed that PeIF2α-mediated ATF4 mRNA translation requires DDX3 as a part of the eIF4F complex.

## Introduction

Cells are constantly subjected to stress ranging from moderate to lethal. To survive stress, cells need to activate pro-survival pathways involving a tight reprogramming of gene expression towards functions that protect cells against stress-induced damage and promote survival. A critical step of stress-induced gene expression remodeling occurs at the level of mRNA translation. Among translational regulatory pathways, phosphorylation of the translation initiation factor eIF2α is the main pathway that is induced during stress^1,2^. When phosphorylated, eIF2α causes a global inhibition of protein synthesis^1,2^ concomitant with the formation of stress granules (SG)^3,4^. These are cytosolic RNA bodies composed of mRNAs and proteins which assemble when general translation initiation is limited, either during stress due to PeIF2α^5^, or upon inactivation of the specific translation initiation eIF4A factor^6^. Stress-induced SG are thus thought to represent a pool of mRNA-proteins complexes stalled in the process of translation initiation, thus contributing to PeIF2α-mediated global translation inhibition^7^. However, while the activation of PeIF2α-SG formation pathway results in a global inhibition of protein synthesis, PeIF2α promotes the preferential translation of specific mRNAs that encode for stress response factors.

ATF4 is a master transcription factor whose induction during ER stress requires phosphorylation of eIF2α^8,9^. PeIF2α drives preferential expression of ATF4 mRNA through a specialised mode of translation re-initiation that relies on the presence of short upstream open reading frames (uORFs) at the 5′ untranslated region (UTR) of the message^10^. In absence of PeIF2α (e.g. in absence of stress), translation re-initiation at uORFs prevents ribosomes from translating the overlapping ATF4 ORF. During ER stress, PeIF2α allows ribosomes to bypass the presence of the inhibitory uORFs, thus re-initiating translation at the main ORF of ATF4 mRNA, which then activates transcription of downstream genes encoding either apoptotic (e.g., ATF3 and CHOP^11^), or adaptive (e.g., chaperones, antioxidants and amino acid synthases)^12–14^ factors. These opposite functions of ATF4 in the cellular stress response depend largely on its expression level linked to its translational regulation by PeIF2α. In cancer, the level of ATF4 mRNA translation induced by PeIF2α is also critical to promote either cell death^11^ or survival^14^ to ER stress generated upon treatment with genotoxic drugs. Mechanisms that regulate PeIF2α-induced ATF4 mRNA translation towards cell survival and cancer cells resistance to drugs remained largely unknown.

We recently described a novel mechanism that regulates eIF2α phosphorylation-mediated ATF4 mRNA translation during ER stress^15^. We found that treatment of cancer cells with ER stress agents exemplified by sorafenib (Sor; a chemotherapeutic agent used to treat hepatocellular carcinoma), induces the formation of SG that sequester a fraction of ATF4 mRNA in a repressed form. Consequently, PeIF2α-mediated lethal overexpression of ATF4 is prevented. The resulting moderate expression of ATF4 in SG-forming cancer cells is necessary for their survival, indicating that maintaining a basal expression of ATF4 is critical for their resistance to genotoxic ER stress^15^. However, factors (e.g. RNA-binding proteins and specific eIFs) responsible for maintaining the translation of ATF4 mRNA allowing the resistance of cells to stress (e.g. resistance of cancer cells to therapeutic agents), remain largely unknown.

eIF4F is a translation initiation complex responsible for the early association of the ribosome with target mRNA during translation initiation. It consists of eIF4E, eIF4GI and eIF4A. While eIF4E is required for the early recognition of mRNA through its interaction with their 5′cap structure^16,17^, eIF4GI serves as a scaffold that recruits eIF4F-bound mRNA to the ribosome through its simultaneous interaction with both eIF4E and eIF4A bound at the 5′end of the mRNA, and with eIF3 bound to ribosomes^16,17^. While this activity of eIF4F in translation initiation is well established, its role in stress-induced translation re-initiation of uORFs-containing mRNAs such as ATF4 mRNA remained however obscure.

DDX3X (here refereed as DDX3) is a nucleocytoplasmic DEAD-box RNA helicase that has been implicated in multiple aspects of gene expression including transcription, mRNA splicing and export^18^. In the cytoplasm, DDX3 can either activate or prevent translation. As a translational repressor, DDX3 was shown to bind and inactivate the translation initiation factor eIF4E, thus repressing translation^19^. As a translational activator, DDX3 was shown to promote translation of viral mRNAs containing structured 5′UTR^20^, though the underlying mechanism remained unknown. It is however noteworthy that DDX3 has been described to bind translation initiation factors, including eIF3^21^ and eIF4GI^22^; the latter interaction is conserved in yeast^23^. Evidence of the role of DDX3 in driving translation of cellular mRNAs was further obtained by combining CLIP-seq with translational assays^24^. These experiments showed that DDX3 preferentially binds 5′ -UTR proximal to the AUG start codon to promote translation of target mRNAs. In this latter study, ATF4 mRNA was recovered as a possible DDX3 interactor. Whether or not DDX3 regulate ATF4 mRNA expression was however not investigated.

Here, we showed that DDX3 positively regulates ATF4 mRNA expression during ER stress induced by Sor treatment of cancer cells. We found that DDX3 drives stress-induced ATF4 mRNA expression at the translational level, and that DDX3-mediated ATF4 translation occurs independently of the PeIF2α−SG pathway. We also demonstrated that during ER stress DDX3 associates with the eIF4F complex, which we found to be required for ATF4 expression. This study thus raised the possibility that the preferential translation of PeIF2α-target mRNAs occurring during ER stress requires the activity of the eIF4F complex-containing DDX3.

## Results

### DDX3 is involved in the expression of ATF4 mRNA during ER stress

**Figure 1:**
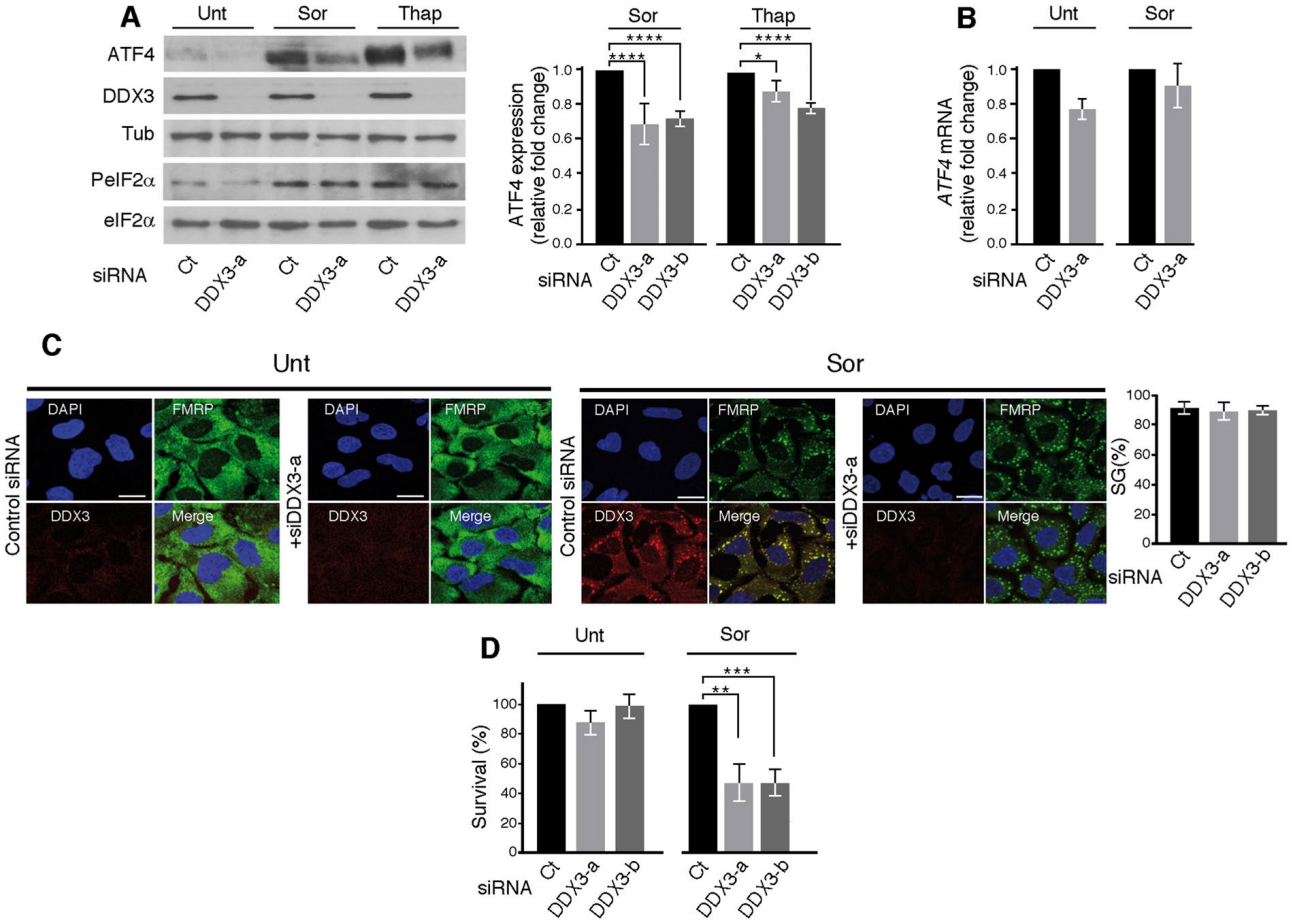
DDX3 is required for ER stress-induced ATF4 expression. (**A**–**D**) Hep3B were treated with either DDX3-a, DDX3-b or control (Ct) siRNAs for ninety-six hours and then incubated with either 10 μM Sor or 100 nM Thap for two hours, as previously described^15^. (**A**) Left panels: Cells were harvested, lysed and proteins materials were analyzed by western blot for the expression of DDX3, ATF4, PeIF2α, the pan-eIF2α, and tubulin (Tub; loading control) using the corresponding antibodies. Right panel: The expression level of ATF4 was estimated by densitometry quantification of the film signal using Image Studio™ Lite Software and standardized against total tubulin. *P ≤ 0.05; ****P ≤ 0.0001 (Student’s *t*-test). The results are representative of at least 3 different experiments. (**B**) Total RNA was isolated and the level of ATF4 mRNA relative to GAPDH mRNA was quantified by real-time q(RT)-PCR using the ΔΔCt method. The presented results are the mean of triplicate measurements, with error bars corresponding to the S.D. (**C**,**D**) Hep3B treated with either DDX3-a, DDX3-b or control (Ct) siRNA were incubated with Sor. (**C**) Cells were processed for immunofluorescence to detect SG using anti-FMRP (green signal) and anti-DDX3 (red signal) antibodies. DAPI stains nuclei. Bars correspond to 20 μm. Representative results from 6 different fields and 3 different experiments containing a total of 1000 cells are shown. Right panel represents the percentage of cells harboring SG (>3 granules/cell). Error bars correspond to the S.D with variations not statistically significant. (**D**) Clonogenic survival assays. Hep3B were incubated with either DDX3-a, -b or control (Ct) siRNAs, then treated with Sor. After trypsinisation, equal numbers of cells were seeded in the absence of drug, and incubated for 10 days. Populations > 20 cells were counted as one surviving colony. Data were calculated as the percentage of surviving colonies relative to the number found in plates corresponding to mock-depleted cells plates. **P ≤ 0.01; ***P ≤ 0.001.

We recently reported the association of ATF4 mRNA with Sor-induced SG^15^. This finding raised the possibility that SG components may be involved in the regulation of ATF4 translation during stress e.g. by promoting the association of ATF4 mRNA with SG, thus preventing its overexpression. Among SG components, DDX3 has been recently identified as a possible ATF4 mRNA interactor^24^; the role of DDX3 in regulating the expression of ATF4 was not investigated. We thus focused on the possible role of DDX3 in the regulation of ATF4 expression upon treatment of the hepatocarcinoma cell line Hep3B with Sor. For this study, we used 10 μM Sor which we have previously defined as a minimal dose required to induce an ER stress characterized by the activation of the PeIF2α-SG pathway^15^. Hep3B were treated with two specific DDX3 siRNAs (a and b) to deplete the protein, incubated with Sor, and then ATF4 expression was monitored by western blot analysis using specific antibodies. Control experiments show that ATF4 was marginally expressed in untreated Hep3B (Fig. 1A), as previously described^15^ and this is consistent with the observed low level of PeIF2α (Fig. 1A). The minimal expression of ATF4 in untreated cells was further prevented in DDX3-depleted cells (Fig. 1A). DDX3 depletion slightly affected the steady-state level of ATF4 mRNA (Fig. 1B), indicating that DDX3 may participate at the transcriptional step of the expression of ATF4 in untreated cells, although we do not exclude a possible post-transcriptional contribution of DDX3.

As we previously reported^15^, treatment of Hep3B with Sor induces ATF4 expression (Fig. 1A), which we have shown to occur mainly at the level of translation through PeIF2α^15^. Control experiments show that Sor treatment indeed induces eIF2α phosphorylation in Hep3B, which was not affected by DDX3 depletion (Fig. 1A). Depletion of DDX3 significantly prevented Sor-induced ATF4 expression in Hep3B (Fig. 1A), and we obtained similar results using HeLa (Supplementary data 1A). While depletion of DDX3 diminished ATF4 level (Fig. 1A), it does not further affect the level of the ATF4 mRNA produced in Sor-treated cells, as compared to untreated cells (Fig. 1B). These results suggest that DDX3 promotes Sor-induced ATF4 expression either at a translational or post-translational level. We obtained similar results (Fig. 1A,B and Supplementary data 1B) upon treatment with thapsigargin (Thap), a classical ER stress inducer. Taken together, our results indicate that DDX3 is required for ATF4 expression induced during ER stress.

As aforementioned, control experiments showed that depletion of DDX3 does not affect eIF2α phosphorylation. This result indicates that PeIF2α is not sufficient by itself to support ATF4 mRNA translation, which further requires the activity of DDX3. However, DDX3 has been previously implicated in the formation of SG, which occurs downstream of PeIF2α^25^. In this study, depletion of DDX3 was shown to prevent SG formation that is induced upon osmotic stress^25^. We thus assessed if DDX3 depletion could affect SG formation upon Sor treatment, thereby affecting ATF4 expression. Hep3B were incubated with either DDX3-a, or DDX3-b siRNAs then treated with Sor to induce SG. The formation of SG was visualised by immunofluorescence using antibodies specific to FMRP, a defined SG marker^26,27^. DDX3 localisation was similarly assessed using the corresponding antibodies. As shown in Fig. 1C, while DDX3 is diffusely distributed in the cytoplasm of untreated cells, it partially co-localized with FMRP in Sor-induced SG. A diffuse and significant signal of DDX3 is also consistently observed in the cytoplasm of Sor-treated cells, indicating that a substantial (~60%) fraction of DDX3 is either not associated with SG or does associates transiently with SG (Fig. 1C and Supplementary data 2). In DDX3 siRNA-treated cells, the immunofluorescence signal corresponding to DDX3 was significantly reduced, attesting for DDX3 depletion (Fig. 1C). Control experiments show that no SG formation is observed in untreated DDX3-depleted cells (Fig. 1C). Upon Sor treatment, DDX3-depleted cells form SG as efficiently as control cells, as visualised and quantified by FMRP staining **(**Fig. 1C **)**. These results showing that DDX3 is not required for SG formation differ from the previously reported role of DDX3 in osmotic stress-induced SG^25^. The source of this discrepancy is currently unknown but may reflect a stress-type specific role of DDX3 in SG formation. Nevertheless, our results show that although DDX3 localises in Sor-induced SG, it is dispensable for their formation (Fig. 1C), indicating that DDX3 promotes ATF4 expression independently of SG formation.

We have previously shown that Sor-induced ATF4 expression which occurs in SG-forming Hep3B is relevant since its elimination by siRNAs abrogated Hep3B cells survival^15^. Our results implicating DDX3 in Sor-induced ATF4 expression prompted us to test if DDX3 contribute to Sor resistance. Hep3B were thus incubated with siDDX3, treated with Sor then collected and analyzed for survival by the clonogenic assay. Control experiments show that depletion of DDX3 per se marginally affects clonogenic survival (Fig. 1D; left panel). Depletion of DDX3 significantly (~2 fold) reduces the clonogenic survival of Sor-treated Hep3B (Fig. 1D; right panel), indicating that DDX3 promotes cell resistance to Sor. DDX3 may contribute to Sor resistance at least in part by maintaining the expression of ATF4, its downstream target (Fig. 1), although we do not have a direct proof for this.

### DDX3 promotes ATF4 expression at the translational level

**Figure 2:**
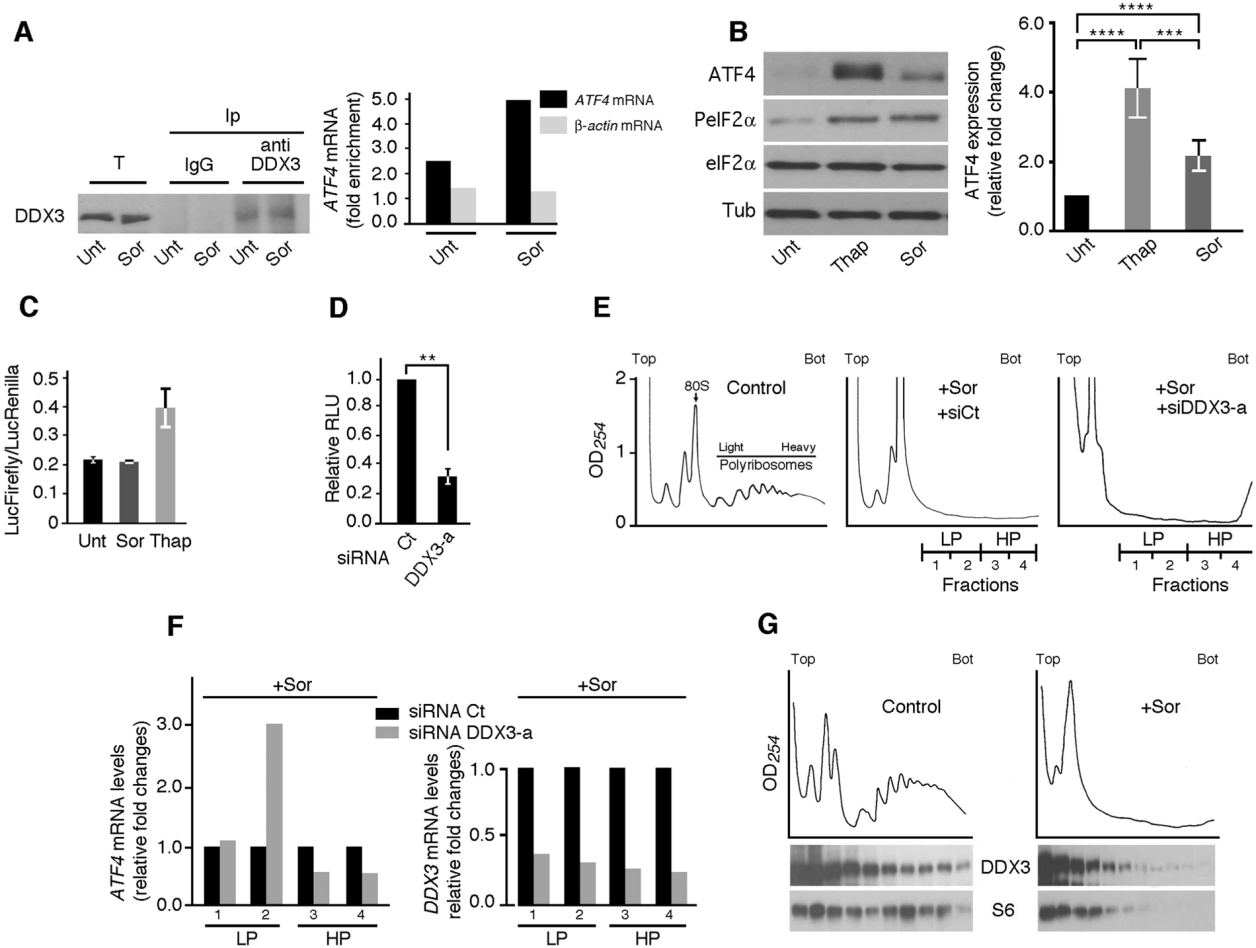
DDX3 regulates ATF4 expression at the posttranscriptional level. (**A**) Immunoprecipitation coupled to qRT-PCR. Hep3B were treated with 10 μM Sor for 2 hours, lysed and their extracts were used to immunoprecipitate DDX3 with anti-DDX3 antibodies and with IgG as a control. mRNAs were isolated from each immunoprecipitate and quantified by qRT-PCR. Left panel: Western blot analysis of total (T) and immunoprecipitated (Ip) proteins using antibodies specific to DDX3. The results are representative of two independent experiments. Right panel: The amounts of ATF4 mRNA and actin mRNA (as control) present in DDX3 precipitate are calculated relative to those in IgG precipitate, then were normalized against GAPDH mRNA. (**B**,**C**) Thap induces ATF4 expression more efficiently than Sor. (**B**) Hep3B were treated with either Sor (10 μM) or Thap (100 nM) then lysed and their protein contents were analyzed by western blot for the expression of ATF4 and tubulin (Tub; loading control) using the corresponding antibodies. P-eIF2α serves a positive control for drugs treatment and the pan-eIF2α is used as control for P-eIF2α. ***P ≤ 0.001; ****P ≤ 0.0001. (**C**,**D**) Translational luciferase assays. (**C**) Hep3B cells are co-transfected with a luciferase expressing vector containing the human ATF4 5′-UTR fused to Firefly luciferase (FLuc) gene, and the control plasmid expressing Renilla luciferase (RLuc). Cells are then treated with either Sor or Thap to induce translation of FLuc whose activity is measured in the cell extracts and expressed relative to RLuc. Error bars correspond to the S.D. (**D**) Hep3B expressing DDX3-a or control (Ct) siRNAs are co-transfected with a luciferase expressing vector containing the human ATF4 5′-UTR fused to FLuc gene, and the control plasmid expressing RLuc. Cells are then treated with Thap to induce translation of FLuc whose activity is measured as above. **P ≤ 0.01. (**E**,**F**) Analysis of the ATF4 mRNA association with polyribosomes. Hep3B were incubated with either DDX3 or control (Ct) siRNAs, then treated with Sor. Cytoplasmic extracts of Sor-treated Hep3B cells were fractionated onto 15% (Top) and 55% (Bot: Bottom) sucrose gradients and their polyribosomes profiles (**E**) were recorded by measuring the OD_254_, as described^27^. LP: light polyribosomes. HP: heavy polyribosomes. (**F**) RNA content was isolated from pooled LP and HP fractions and associated ATF4 mRNA was quantified by qRT-PCR using the ΔΔCt method. ATF4 mRNA levels were normalized against 18 s ribosomal RNA and expressed as indicated (left panels). DDX3 mRNA present in LP and HP was similarly quantified and serves as an additional control for DDX3 mRNA knockdown (right panel). The results are representative of two independent experiments. (**G**) Top panels: cytoplasmic extracts prepared from either untreated or Sor-treated Hep3B were fractionated through 15–55% (w/v) sucrose density gradients and their polyribosomes profiles were monitored as above. Bottom panels: Western blot analysis of the collected fractions for the distribution of DDX3 and as a control S6 protein using specific antibodies. The results are representative of two independent experiments.

Based on the results described above, mainly that DDX3 depletion prevented Sor-induced ATF4 expression without affecting the RNA level of ATF4 (Fig. 1B), we postulated a role of DDX3 in driving translation of ATF4 mRNA. This assumption is consistent with our localisation studies showing that a significant fraction of DDX3 is diffusely distributed in the cytoplasm of Sor-treated cells (Fig. 1C and Supplementary data 2), which may support PeIF2α-driven ATF4 translation (Fig. 1A). To investigate the possibility that DDX3 affects ATF4 expression at its translation level we first tested if DDX3 forms a complex with the ATF4 mRNA in Sor-treated Hep3B. Immunoprecipitation of DDX3 followed by quantitative RT-PCR (qRT-PCR) of bound mRNAs supported the association of ATF4 mRNA with DDX3-containing complexes (Fig. 2A). As described above, translation of ATF4 mRNA during Sor treatment occurs via a mechanism that requires phosphorylation of eIF2α^15^. PeIF2α drives preferential translation of ATF4 mRNA through a specialised mode of translation re-initiation that relies on the presence of short upstream open reading frames (uORFs) at the 5′UTR of the mRNA^10^. To test if DDX3 is involved in PeIF2α-mediated ATF4 translation initiation, we devised the standard luciferase translational reporter assay that is widely used to assess translation of uORFs-harboring mRNAs^28^. Briefly, Hep3B expressing either DDX3 or control siRNAs are co-transfected with a luciferase expressing vector containing the human ATF4 5′UTR fused to Firefly luciferase (FLuc) gene, and the control plasmid expressing Renilla luciferase (RLuc). For these experiments, we favored Thap that induced ATF4 expression more efficiently than Sor (Fig. 2B) and therefore is effective in inducing FLuc (Fig. 2C). DDX3-depleted cells are thus treated with Thap to induce translation of FLuc whose activity is measured in the cell extracts and expressed relative to RLuc. Using this reporter translation re-initiation assay, we found that depletion of DDX3 with either DDX3-a (Fig. 2D) or -b (data not shown) resulted in a significant (~5 fold) reduction of FLuc activity in Thap-treated Hep3B, supporting a role of DDX3 in promoting ATF4 translation initiation during ER stress.

We then sought to confirm our results using the *in vivo* polyribosome translation assays. This approach relies on the purification of polyribosomes on sucrose gradients followed by monitoring the distribution of mRNAs based on the number of bound ribosomes, which reflects their translational efficiency^29^. Efficiently translated mRNAs are generally those associated with heavy translating polyribosomes (>4 ribosomes) corresponding to fractions sedimenting toward the bottom of the sucrose gradient. We have previously used this approach to profile polyribosomes from Sor-treated cells and quantify their bound ATF4 mRNA^15^. In that study, we showed that Sor treatment of Hep3B induced a major loss of polyribosomes, attesting for an inhibition of general translation^15^. qRT-PCR of polyribosomal-associated mRNAs showed however that a fraction of ATF4 mRNA associates with the remained polyribosomes in Sor-treated cells, which is consistent with our data showing that Sor treatment induces translation of the ATF4 mRNA^15^. If DDX3 is required for promoting ATF4 mRNA translation in Sor-treated cells, then its depletion should affect the association of ATF4 mRNA with polyribosomes. As mentioned above, ATF4 mRNA is marginally translated in unstressed Hep3B^15^ (see also Fig. 1A, and Supplementary data 1), which precludes the analysis of its polyribosomal distribution. Thus, we focused our analysis on measuring the polyribosomal distribution of the ATF4 mRNA expressed in Sor-treated Hep3B upon DDX3 depletion. Hep3B were incubated with DDX3-a siRNA to deplete DDX3, and treated with Sor. Polyribosomes were then purified, profiled and bound ATF4 mRNA was assessed by qRT-PCR. As previously described^15^, Sor treatment induced a significant loss of polyribosomes (Fig. 2E). Sor-induced loss of polyribosomes is similar between mock- and DDX3-depleted Hep3B (Fig. 2E). qRT-PCR of polyribosomes-associated mRNAs showed that depletion of DDX3 (Fig. 2F; right panel) reduced the levels of ATF4 mRNA in fractions corresponding to heavy translating polyribosomes (HP; fractions 3–4) of Sor-treated cells (Fig. 2F; left panel). Loss of mRNAs from translating polyribosomes, e.g. during ER stress, is generally an indication of the inhibition of their translation at the level of the initiation step^29^. Thus, our results showing that depletion of DDX3 disrupted the association of ATF4 mRNA with HP further indicate that DDX3 is involved in its translation most-likely at its initiation step (Fig. 2F). Surprisingly, depletion of DDX3 also resulted in an enrichment of the ATF4 mRNA in fractions corresponding to light polyribosomes (LP; fraction 2) (Fig. 2F). Moderately translated or translationally stalled mRNAs are generally associated with light polyribosomes (<4 ribosomes) that sediment at the middle of the sucrose gradient. Thus, the accumulation of ATF4 mRNA in LP in DDX3-depleted cells suggests that in absence of DDX3, translation of the ATF4 mRNA may be stalled at its elongation step. Collectively, our data strongly support a role of DDX3 in promoting ER stress-induced translation of ATF4 mRNA at its initiation step, and possibly also at its elongation step.

If DDX3 is involved at both initiation and elongation steps of the ATF4 mRNA translation, then it should associate with both translation initiation complexes and translating polyribosomes. Cytoplasmic extracts were prepared from both untreated and Sor-treated cells and processed through sucrose density gradients followed by separation of translation initiation complexes and polyribosomes fractions (Fig. 2G; top panels). The recorded normal polyribosome profile is validated by western blot analysis of isolated fractions using anti-S6 antibodies (Fig. 2G, bottom panels). Western blot analysis of the separated fractions showed that DDX3 is distributed throughout the gradient (Fig. 2G, left middle panel), indicating that DDX3 associates with both translation initiation complexes that sediment at the top of the sucrose gradient, and translating polyribosomes sedimenting at both the middle and bottom of the gradient. Our data showing that DDX3 is greatly associated with polyribosomes in untreated cells (Fig. 2G, left middle panel), are consistent with recent results implicating DDX3 in translation regulation of a wide range of mRNAs under normal growth conditions^24^. Despite the massive loss of polyribosomes in Sor-treated cells (Fig. 2G, right top panel), DDX3 is still detected, albeit slightly, in fractions corresponding to the residual polyribosomes (Fig. 2G, right middle panel). The distribution of DDX3 is however similar to the distribution of the control S6 protein at the polyribosomes fractions (Fig. 2G, right bottom panel), which further supports a possible role of DDX3 in translation elongation of the ATF4 mRNA in Sor-treated cells. Although the association of DDX3 with polyribosomes is minimal in Sor-treated cells as compared to untreated cells (Fig. 2G; middle panels), it is relevant as it promotes translation of ATF4 (Figs. 1A and 2D–F) to a level that is sufficient for cell resistance to Sor (Fig. 1D). Finally, DDX3 also distributed in fractions corresponding to translation initiation complexes in Sor-treated cells (Fig. 2G, right bottom panel), which is consistent with its role in driving translation initiation of the ATF4 mRNA (Fig. 2D–F).

### DDX3 is a component of the eIF4F translation initiation complex that drives translation of the ATF4 mRNA upon ER stress

Although it is well established that stress-mediated translation of the ATF4 mRNA is regulated mainly at its initiation step via PeIF2α^8–10^, it is still largely unknown if this PeIF2α-mediated ATF4 mRNA translation involves the activity of specific translation initiation factors. *In vitro* studies showed that re-initiation after a short uORF in rabbit reticulocyte lysates requires eIF4F translation initiation complexes^30^. Further, more yeast studies implicated eIF4GI, the component of eIF4F, in stress-mediated translation of uORFs-containing mRNAs^31^. As far as we know however, the role of mammalian eIF4GI in the expression of uORFs-containing mRNAs including ATF4 mRNA during stress is currently unknown. eIF4GI being a partner of DDX3^22^, we speculated the possibility that both proteins are part of a common eIF4F complex that promotes Sor-induced translation of the ATF4 mRNA.

**Figure 3:**
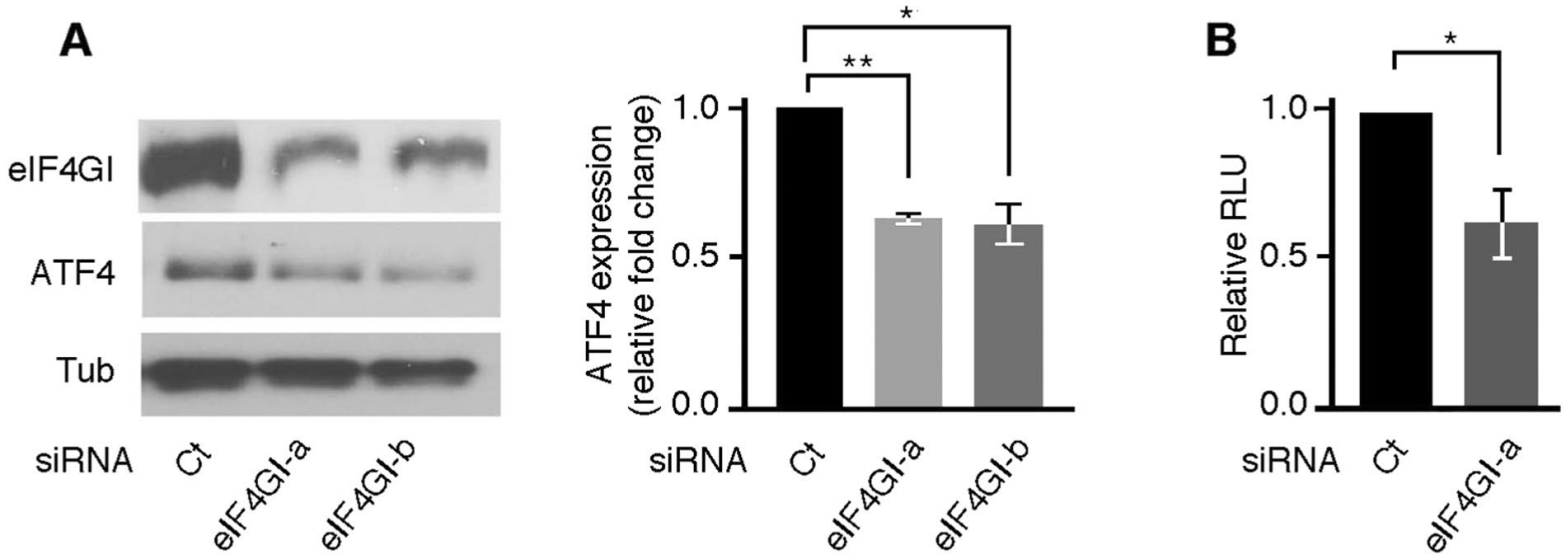
eIF4GI is required for ATF4 expression upon stress. (**A**) Hep3B were incubated with either eIF4GI-a, eIF4GI-b or control (Ct) siRNAs, treated with Sor, then lysed. Proteins content was analyzed for the expression of ATF4, eIF4GI, and tubulin (loading control) using the corresponding antibodies. Right histogram: The expression level of ATF4 was estimated as in Fig. 1. *P ≤ 0.05; **P ≤ 0.01 (**B**) Translational luciferase assays. Hep3B expressing either eIF4GI-a or a control (Ct) siRNA are co-transfected with a luciferase expressing vector containing the human ATF4 5′-UTR fused to FLuc gene, and the control plasmid expressing RLuc. Cells are then treated with Thap to induce translation of FLuc whose activity is measured as above. *P ≤ 0.05.

We first assessed the role of eIF4GI in driving ATF4 translation. Hep3B were treated with two specific eIF4GI siRNAs, incubated with Sor then ATF4 expression was assessed as above. We found that depletion of eIF4GI significantly abrogated ATF4 expression (Fig. 3A), indicating that eIF4GI participates in the process of translation re-initiation of ATF4 mRNA during ER stress. We further assessed the role of eIF4GI in that process using luciferase reporter assays as described above. We found that depletion of eIF4GI significantly reduced FLuc activity upon Thap treatment (Fig. 3B). Depletion of eIF4GI had however less effect on FLuc activity than DDX3 depletion (compare Fig. 3B with Fig. 2D). FLuc activity that occurs in eIF4GI-depleted cells may be due to the activity of other eIF4G isoforms such as eIF4GII. Alternatively, residual eIF4GI that is still expressed in eIF4GI siRNA-treated cells may be sufficient to drive the observed FLuc activity. In any case, these results supported a novel role of eIF4GI, the eIF4F component, in ER stress-induced expression of ATF4, most-likely at the level of its translation initiation step.

**Figure 4:**
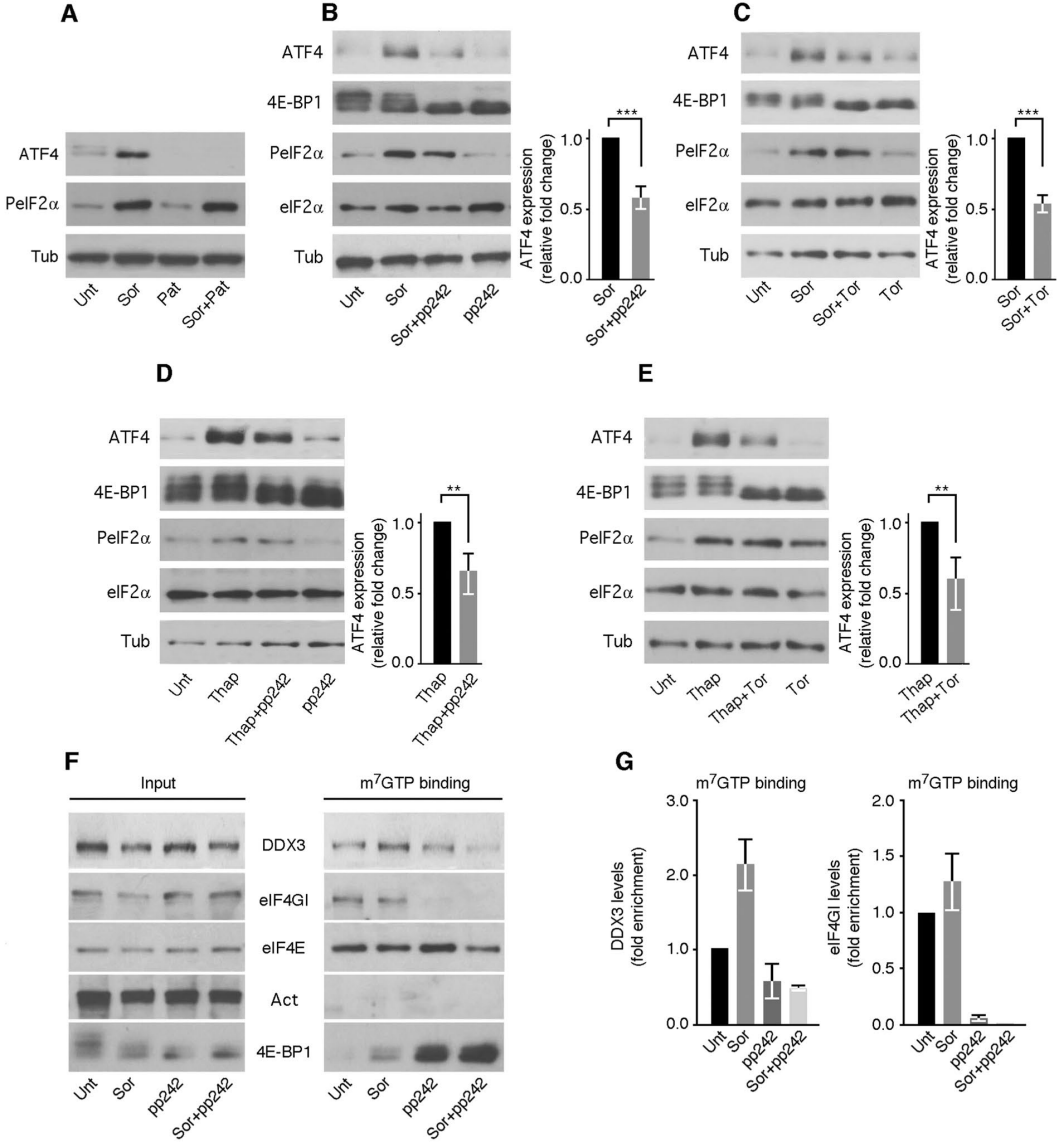
DDX3 is a component of the eIF4F complex that is required for Sor-and Thap-induced ATF4 expression. (**A**–**C**) Interfering with the eIF4F activity prevents Sor-induced ATF4 expression. (**A**) Hep3B treated with either Sor (10 μM), pateamine A (Pat; 100 nM) or both were collected and their protein contents analyzed for the expression of ATF4, and PeIF2α. Τubulin serves as a loading control. (**B**,**C**) Hep3B were treated with either Sor (10 μM), pp242 (2.5 μM), Torin 1 (Tor; 0.1 μM), or the combination of the drugs as indicated. Cells were then lysed and their proteins content analyzed for the expression of ATF4 and PeIF2α. Tub is used as a loading control for ATF4 expression and the pan-eIF2α serves as a control for P-eIF2α. Hypophosphorylation of 4E-BP1 (lower band) serves as a control for mTORC1 inactivation with pp242. Quantification of ATF4 expression is shown in the right graphs. Error bars indicate S.D. ***P ≤ 0.001 (Student’s *t*-tests). (**D**,**E**) Hep3B were treated with either Thap (100 nM), pp242 (2.5 μM), Tor (0.1 μM), or the combination of the drugs as indicated. Cells were then collected and their protein contents analyzed as above. Quantifications of ATF4 expression are shown in the right graphs. Error bars indicate S.D. **P ≤ 0.01 (Student’s *t*-tests). (**F**,**G**) DDX3 is a component of the eIF4F complex. Cap-binding assays. Hep3B were treated with Sor, pp242 or both as above. (**F**) Cells were harvested and their lysates were quantified. Equal amounts of lysates were incubated with the cap analogue m^7^GTP coupled to Sepharose beads. Bound eIF4F complexes were eluted (m^7^GTP binding) and analyzed by Western blot using specific antibodies. Actin serves as negative control for cap-binding. 4E-BP1 serves as a positive (Lanes Sor + pp242 and pp242) control for cap binding. (**G**) DDX3 and eIF4GI enrichments in the m^7^GTP binding eluates recovered from Sor-, pp242-, and Sor + pp242-treated cells were estimated by densitometry quantitation of the film signal using Image Studio™ Lite Software, standardized to respective DDX3 or eIF4GI levels in the input, and then expressed relative to the corresponding protein level in the m^7^GTP binding eluate of untreated cells. Error bars correspond to the S.D.

We thus sought to further extend our results testing if ER stress-induced ATF4 translation involves the formation of eIF4F complex. To interfere with eIF4F activity, we first used pateamine A, which is known to inhibit translation initiation by targeting eIF4A^32^. We found that treatment of either Hep3B (Fig. 4A), Huh-7 (Supplementary data 3A) or HeLa (data not shown) with pateamine A abrogated Sor-induced ATF4 expression without affecting the phosphorylation of eIF2α. These results suggest an essential role of eIF4F in Sor-induced ATF4 expression, which we further assessed by blocking its formation using inhibitors that disrupt the interaction between eIF4E and eIF4GI. The interaction between eIF4GI and its partner eIF4E is regulated mainly via the mammalian target of rapamycin complex 1 (mTORC1). By phosphorylating 4E-BP1, a molecular competitor of eIF4GI^33^, mTORC1 promotes eIF4E-4GI interaction, therefore activating translation initiation^17,34^. Among mTORC1 inhibitors recently developed, pp242^35,36^ potently inactivate mTORC1 and prevents formation of downstream eIF4F complex^37,38^. Using pp242, we found that inactivating the eIF4F complex significantly abrogates ATF4 expression in Sor-treated Hep3B without affecting the level of PeIF2α (Fig. 4B). Control experiments show that addition of pp242 induced hypophosphorylation of 4E-BP1, thus marking the inactivation of the eIF4F complex in Sor-treated Hep3B. We obtained similar results (Fig. 4C) using Torin 1 (Tor), which like pp242 is a potent inhibitor of mTORC1^37,38^. ATF4 expression was also blocked in Huh-7 (Supplementary data 3B), HeLa and MEFs (data not shown) that are treated with either pp242 or Tor. Treatment of both Hep3B (Fig. 4D,E) and Huh-7 (Supplementary data 3C) with either pp242 or Tor also prevented ATF4 expression that is induced with Thap, further indicating a general role of the eIF4F complex in ER stress-induced ATF4 expression.

So far, our results showed that both eIF4GI (Fig. 3) and its DDX3 (Figs 1 and 2) partner promote Sor-induced ATF4 translation initiation, raising the possibility that eIF4GI and DDX3 may be part of a common eIF4F translation initiation complex required for ATF4 translation (Fig. 4A–E). To investigate this possibility, we used the cap-binding assay, a reliable *in vitro* test^39^ that was successfully used to characterize the formation of the eIF4F complex under various growth conditions^40–43^. Hep3B cell lysates were incubated with the cap analogue m^7^GTP bound to Sepharose beads. The cap-binding protein eIF4E along with its bound partners were then eluted and analyzed by Western blotting. As expected, both eIF4E and its partner eIF4GI were recovered in the eluates of lysates prepared from either untreated or Sor-treated cells (Fig. 4F). DDX3 was also recovered in those eluates (Fig. 4F) attesting for its interaction with the eIF4F complex. Thus, DDX3 is a component of the eIF4F complex as analyzed here by the cap-binding assay and in keeping with mass spectrometry data analysis of cap-binding complexes^44^. Our quantifications show however an enhanced association of DDX3 with cap-binding complexes in Sor-treated cells (Fig. 4G). We thus tested if disrupting eIF4F complex in Sor-treated cells by addition of pp242 affects DDX3 binding. Control experiments showed that treatment of cells with pp242 resulted in a loss of eIF4GI (Fig. 4F,G) with a concomitant recovery of its 4E-BP1 competitor (Fig. 4F) in the eluates. DDX3 was also lost in the eluates of pp242-treated cells (Fig. 4F,G). Our results (Fig. 4F,G) indicate that disrupting eIF4E-4GI interaction with pp242 affects the association of DDX3 with the cap-binding complexes required for ATF4 expression (Fig. 4A–E). Altogether, our data suggest that DDX3 promotes ATF4 mRNA translation as a functional component of the eIF4F complex. DDX3 was also marginally recovered in the eluates corresponding to extracts that are prepared from Hep3B treated with both Sor and pp242 and which are also lacking eIF4GI (Fig. 4F,G). Thus pp242 induces displacement of both DDX3 and eIF4GI from the eIF4F complex in Sor-treated cells. We obtained similar results upon Thap treatment (Supplementary data 4), further validating the association between DDX3 and the eIF4F complex during ER stress, which can be displaced by mTORC1 inhibitors. The strong correlation between the displacement of both DDX3 and eIF4GI from the eIF4F complex (Fig. 4F,G) and the inefficient induced expression of ATF4 in pp242-treated cells upon exposure to either Sor (Fig. 4B) or Thap (Fig. 4D) supports our assumption that eIF4GI and its partner DDX3 are two functional partners of the eIF4F complex required for ER stress-mediated translation of the ATF4 mRNA.

## Discussion

Our study added new mechanistic details of the PeIF2α-mediated ATF4 translation mode. We found that PeIF2α-driven ATF4 mRNA translation initiation in Sor-treated cells involves DDX3. The identification of DDX3 as a component of the eIF4F complex in Sor-treated cells suggests that it promotes ATF4 mRNA translation through its association with eIF4F components, including its partner eIF4GI. In agreement, displacement of DDX3 from the eIF4F complex lacking eIF4GI prevented Sor-induced ATF4 expression; we obtained similar results through eIF4GI depletion. The role of DDX3 in driving ATF4 mRNA translation is not restricted to Sor as we obtained similar results in cells treated with Thap, which like Sor induces an ER stress.

Both yeast and mammalian studies indicated that DDX3 plays an early role in translation initiation. Studies in yeast suggested that DDX3, through its unwinding activity of the secondary structure of the 5′UTR, promotes translation by assisting 40S scanning of the mRNA^45^. In contrast to yeast DDX3, the role of mammalian DDX3 in translation seems to be restricted to specific target mRNAs^20,46^. In one study^20^, depletion of DDX3 was shown to impair translation of reporter mRNAs that have long and structured 5′UTR. This suggested that DDX3 may selectively promote translation of target mRNAs that have extensive secondary structures. Validation of this assumption was recently obtained through CLIP-seq experiments showing preferential binding of DDX3 to the 5′UTR of specific mRNAs, thereby promoting their translation^47^. This result contrasts however with those showing that enforced expression of DDX3 contributes, albeit modestly, to arsenite-mediated general translation repression. This was attributed in part to the DDX3 association with SG^24^, although direct evidence is still lacking. Our results do not exclude the possibility that DDX3 may also contribute to ER stress-mediated general translation inhibition while maintaining translation of specific mRNAs including ATF4 mRNA. In keeping with a possible dual translational role of DDX3 during ER stress, we found that DDX3 localises at both SG where mRNAs are translationally repressed, and in the diffuse cytoplasm (Fig. 1C and supplementary data 2) where its supports ATF4 mRNA translation (Fig. 2). Ribosome or polyribosome profiling of mRNAs in DDX3-depleted cells may help identify mRNAs whose translation is regulated during ER stress in a DDX3-dependant manner.

Both luciferase (Fig. 2D) and polyribosomes assays (Fig. 2E–G) support a role of DDX3 in driving translation initiation of ATF4 mRNA during ER stress, which may involve its interaction with the eIF4F complex. Indeed, pharmacological inactivation of the eIF4F complex using either pateamine (Fig. 4A), pp242 (Fig. 4B and D) or Tor (Fig. 4C and E) suppressed both Sor- and Thap-induced ATF4 expression. As far as we know, this result provided the first evidence that eIF4F is required for ATF4 mRNA translation during ER stress. Our results showing that disruption of the eIF4F complex with either pp242 or Tor prevents ATF4 expression upon treatment with either Sor (Fig. 4B and C) or Thap (Fig. 4D and E) are in agreement with a recent study implicating mTORC1 in ATF4 mRNA translation. In that study^48^, however, Ben-Sahra *et al*. showed that treatment of cells with rapamycin (a partial inhibitor of the translational eIF4F arm of mTORC1) prevented growth factor-induced ATF4 expression, but not the upregulation of ATF4 upon amino acid starvation or exposure to the ER stress-inducing agent tunicamycin. This suggests that while partial inhibition of the translational axis of mTORC1 with rapamycin is sufficient to prevent ATF4 expression that is induced independently of eIF2α (e.g. through growth factors), it is insufficient to abrogate PeIF2α-induced ATF4 expression. As aforementioned, rapamycin inhibits mTOR enzymatic activity indirectly and thus partially affects mTORC1-mediated translation. In contrast, both pp242 and Tor bind directly to the mTOR kinase domain and potently suppresses its activity^36,49^. As a consequence, pp242 and Tor are very efficient in inhibiting the activity of the mTORC1-driven eIF4F^36,49^, which reflects our results showing that inhibition of mTORC1 with either pp242 or Tor significantly prevented ATF4 expression during ER stress (Fig. 4 and Supplementary data 3). Importantly, our data extended the results of Ben-Sahra *et al*. by (i) implicating mTORC1 in PeIF2α-driven ATF4 expression, and (ii) providing the first evidence of the role of the mTORC1-downstream mammalian eIF4F complex in ER stress-induced ATF4 mRNA translation (Figs 3 and 4). While our study was under revision, a paper was published implicating 4E-BP1, the inhibitor of eIF4F complex formation, in downregulating ATF4 expression^50^. In this study, the authors showed that 4E-BP1 represses ATF4 translation independently of PeIF2α, which we further extended by revealing that the eIF4F complex, the target of 4E-BP1, is required for PeIF2α-mediated ATF4 expression. Through cap-binding assays, we further confirmed DDX3 as a component of the eIF4F complex (Fig. 4F,G). We found that DDX3 binding to the eIF4F complex was however altered upon addition of pp242 that also displaces eIF4GI from the complex (Fig. 4F,G). These results are consistent with a recent study showing mTOR-regulated association of DDX3 with cap-binding complexes^44^. DDX3 binding to the eIF4F complex was enhanced in Sor-treated cells; an interaction that was also disrupted upon removal of eIF4GI by addition of pp242 (Fig. 4F,G and Supplementary data 4). The strong correlation between the displacement of both DDX3 and eIF4GI from the eIF4F complex (Fig. 4F,G) and the inefficient Sor- and Thap-induced expression of ATF4 in either pp242-treated cells (Fig. 4), Tor-treated cells (Fig. 4), DDX3- (Fig. 1 and Supplementary data 1), or in eIF4GI-depleted cells (Fig. 3) supports our assumption that DDX3 is a functional eIF4GI partner of the eIF4F complex required for ER stress-induced translation of the ATF4 mRNA.

How DDX3 and eIF4F cooperate to promote PeIF2α-mediated ATF4 mRNA translation during ER stress is still unclear. It is worthy to note however that both DDX3 (this study) and the eIF4F complex^15^ partially localise in Sor-induced SG and in the cytoplasm (Fig. 1C). The localisation of eIF4F components in SG should limit the eIF4F complex that is required for translation in the cytoplasm. We speculate the possibility that under such limiting conditions, cytoplasmic DDX3 recruits a fraction of the eIF4F complex to its bound ATF4 mRNA therefore forming a translation initiation competent complex and enhancing its translation initiation. A recent study^22^ proposed that DDX3 facilitates the association of the eIF4F complex with the 5′end of viral RNAs by unwinding secondary structures located near the cap. Once eIF4F is stabilised over the cap structure, eIF4A through its helicase activity helps scanning the 5′UTR of the message by the ribosome. We speculate that DDX3 may be similarly critical for anchoring the DDX3-containing eIF4F complex to the cap structure of ATF4 mRNA to allow its translation initiation. This hypothesis will be addressed in a follow-up study.

Our study has also implications in cancer research. Both ATF4^14,51^ and DDX3^52^ have been shown to promote cancer development and resistance to therapeutic treatment. We have previously shown that direct inactivation of ATF4 expression with siRNAs abrogates hepatocarcinoma cells resistance to Sor^15^. Here we show that downregulating DDX3, the upstream regulator of ATF4 mRNA translation, also sensitizes hepatocarcinoma cells to Sor (Fig. 1D), thus identifying DDX3 as a novel factor which may be targeted to prevent resistance to Sor.

## Material and Methods

### Cell culture

Hep3B and Huh-7 were received from Dr. M. Bilodeau (Université de Montréal). HeLa cervical cancer cells were purchased from American Type Culture Collection (ATCC). All cell lines were maintained in Dulbecco’s modified Eagles’ medium (DMEM) (Wisent) supplemented with 5% heat-inactivated fetal bovine serum (FBS; Wisent), penicillin and streptomycin at 37 °C in 5% CO_2_.

### Drug treatments

Sorafenib and both mTOR inhibitors pp242 and Torin 1 were purchased from Selleck Chemicals. Thapsigargin was purchased from Sigma and Pateamine A was kindly provided by Dr. J. Pelletier (McGill University). Sorafenib, pp242 and Torin 1 were dissolved in DMSO as a 10 mM, Thapsigargin as a 1 mM, and Pateamine as a 100 µM stock solution, aliquoted and stored at −80 °C. For drugs treatment, cells were plated to reach a confluency of ~80–90% the day of the treatment. The media was changed with fresh media 2 hours before treatment.

### Antibodies

Phospho-specific anti-eIF2α, anti-eIF2α, anti-eIF4GI, anti-4EBP1 were obtained from Cell Signaling Technology (Beverly, MA). Anti-DDX3 and anti-tubulin antibodies were purchased from Abcam, and anti-ATF4 antibody from Santa Cruz Biotech. Anti-eIF4E was obtained from BD Bioscience. Anti-HuR and anti-FMRP antibodies were previously described^15,53^.

### Immunofluorescence analyses

For immunofluorescence experiments, all fixation, permeabilisation and staining procedures were performed according to the previously described protocol^40^. Immunostainings were visualized using LSM 700 confocal laser scanning microscope (Zeiss) controlled with ZEN 2009 software for image acquisition and analysis, as described before^40^.

### siRNAs and siRNA transfection

siRNAs were purshased from Dharmacon (Lafayette, CO). siRNA transfections were performed, using Hiperfect reagent (Qiagen) as previously described^40^.

The sequences of siRNAs used in this study are:

siRNA-DDX3-a: sense: 5′ G.C.A.A.A.U.A.C.U.U.G.G.U.G.U.U.A.G.A 3′

siRNA-DDX3-b: sense: 5′ G.G.A.G.A.A.A.U.U.A.U.C.A.U.G.G.G.A.A 3′

siRNA-eIF4GI-a: sense: 5′ U.G.A.G.A.A.A.G.G.A.G.G.A.G.A.G.G.A.A 3′

siRNA-eIF4GI-b: sense: 5′ G.G.G.C.U.U.A.G.C.U.G.G.A.A.G.G.A.A.U 3′

### Cap Binding assay

The cap-binding assay was done as previously described^40^. Briefly, cells were harvested and lysed in buffer A (50 mM Tris-HCl [pH 7.4], 100 mM NaCl, 1 mM EDTA, and protease inhibitors [Roche] and supplemented with 0.5% NP-40). Lysates were then incubated with the mRNA cap analog m^7^GTP-Sepharose (GE Healthcare) in buffer A. The m^7^GTP-Sepharose-bound proteins were washed with buffer A, and cap-binding complexes were then eluted with SDS loading buffer, resolved by SDS-PAGE, and analyzed by western blotting.

### RNA immunoprecipitation

Cells were lysed in buffer B (50 mM Tris–HCl, pH 7.4, 150 mM NaCl, 1 mM MgCl2, 0.5% NP-40) supplemented with 0.25 mM PMSF, 0.5 mM DTT, protease inhibitors cocktail (Roche), and 40 U/ml RNase inhibitor (Invitrogen). Lysates were then clarified by centrifugation at 10.000 rpm and the resulting supernatant used for immunoprecipitation as described^54^. Following immunoprecipitation, proteins were digested by proteinase K and RNA was isolated using the phenol extraction method. RNA was then precipitated before been resuspended in RNase-free H_2_O.

### DNA transfection and Luciferase reporter assay

Forty-eight hours following siRNA transfection, Hep3B were transfected with a mixture of pRL-TK-Renilla, and the p5′UTR-ATF4-FLUC plasmids using a Transfection reagent kit (Qiagen). Forty-eight hours later, cells were resuspended in passive lysis buffer (Promega) and the firefly and *Renilla* luciferase activities were measured using a dual-luciferase reporter assay system (#E1960; Promega) according to the manufacturer’s instructions. Luciferase activity was measured by a Tecan Infinite F200 multimode microplate reader.

### Polyribosomal profiles and analyses of polyribosomal-associated mRNA

To prepare polyribosomes, we used the same protocol we previously described^15,29^. Briefly, cells grown in 100-mm tissue culture (~ 80–90% confluence) were treated with sorafenib, then lysed with polyribosomal buffer (20 mM Tris, pH 7.4, 150 mM NaCl, 1.25 mM MgCl_2_, 8 U/ml RNase inhibitor [Invitrogen], protease inhibitor cocktail [Complete; Roche], 1 mM DTT, and Nonidet P-40 at a final concentration of 1%. Extracts were clarified by centrifugation and the resulting cytoplasmic extracts were loaded on 15–55% (w/v) linear sucrose gradient for sedimentation by ultracentrifugation. RNA-proteins complexes of individual isolated fractions were ethanol precipitated. For protein analysis, proteins were resuspended in SDS-PAGE sample buffer. For RNA analysis, precipitated complexes were resuspended in RNase-free H_2_O, pooled and processed for RNA extraction and analysis as described below.

### Quantitative Real-time PCR analysis

Total RNA was extracted with the Trizol reagent (Life Technology) and polyribosomal RNA was prepared by phenol-chloroform extraction. RNA was then reverse transcribed using the Quantitect Reverse Transcriptase kit (Qiagen). Each reaction contained 40 ng of RNA, 2 μl of genomic DNA Wipeout Buffer 7X, 4 μl of Quantiscript RT Buffer 5X, 1 μl of RT Primer Mix and 1 μl of Quantiscript Reverse Transcriptase. Real-time PCR reactions were prepared using the Power SYBRH Green PCR Master mix (Applied Biosystems, Streetsville, ON, Canada) in a total volume of 15 μl: 7.5 μl of PCR Master Mix, 0.22 μl of forward primer at 10 μM, 0.22 μl of reverse primer at 10 μM, 1.06 μl of deionized (Milli-Q grade) water and 6 μl of cDNA.

The primer pair for ATF4 mRNA was: 5′-CACTAGGTACCGCCAGAAGA-3′ (forward), and 5′-AATCCGCCCTCTCTTTTAGA-3′ (reverse). For B-Actin mRNA, the primer pair used was: 5′-CCCTGGAGAAGAGCTACGAG-3′ (forward), and 5′-AGGTAGTTTCGTGGATGCCA-3′ (reverse). The primer pair for GAPDH mRNA was: 5′-ACC CAC TCC TCC ACC TTT G-3′(forward) and 5′-CCA CCA CCC TGT TGC TGT-3′, and for the DDX3 mRNA was: 5′-TTCGAGACTTGGAACGTGGA-3′(forward) and 5′-ACGAATCTGAGGCTCAAACC-3′ (reverse) as oligonucleotides. For 18 s rRNA, the following primer pair was used: 5′-AAACGGCTACCACATCCAAG-3′ (forward) and 5′-CCTCCAATGGATCCTCGTTA-3′ (reverse).

### Clonogenic assays

This assay was done essentially as described^15^. In brief, cells were trypsinized, counted, replated in 6-well plates at 1000 cells/well, and incubated for 8–10 days. Cells are then washed with PBS, stained (0.1% (w/v) crystal violet in a 0.0037% (v/v) formaldehyde solution in PBS), and isolated colonies were counted.

## Electronic supplementary material

Supplementary data
