# Supplementary material for: DDX3 regulates endoplasmic reticulum stress-induced ATF4 expression
Source: Sci Rep. 2017 Oct 23;7:13832. doi: 10.1038/s41598-017-14262-7 (PMC5653821; doi:10.1038/s41598-017-14262-7)
Supplement: Supplementary file 1 — Supplementary data [file 41598_2017_14262_MOESM1_ESM.pdf]

Supplementary data

DDX3 regulates endoplasmic reticulum stress-induced ATF4 expression

Pauline Adjibade[1], Valérie Grenier St-Sauveur[1*], Jonathan Bergeman[1], Marc-Etienne Huot[1], Edouard W. Khandjian[2], and Rachid Mazroui[1**].

Supplementary data 1. (A) HeLa were treated with either DDX3-a, or control (Ct) siRNAs then incubated with Sor as described in figure 1. Cells were harvested, lysed and protein materials were analyzed by western blot for the expression of DDX3, ATF4 and HuR (loading control) using the corresponding antibodies. The indicated expression level of ATF4 was estimated by densitometry quantitation of the film signal using Image Studio™ Lite Software and standardized against total HuR. (B) Hep3B were treated with either DDX3-a, or control siRNA, then incubated with Thap. Total RNA was isolated and the level of ATF4 mRNA relative to GAPDH mRNA was quantified by q(RT)-PCR

Supplementary data 2. Densitometry quantification of DDX3 signal (red) shown in figure 1C with Adobe Photoshop software. The numbers of pixels and mean intensities were measured for individually selected regions (Total, diffuse cytoplasm referred here as cytoplasm, SG, and background). The mean intensity was multiplied by the number of pixels for each selected region. The absolute intensity of the background region was subtracted to the obtained absolute intensity of each selected region. Relative intensities were then calculated and correspond to the absolute intensities relative to the intensity of the reference. Results are expressed as the mean of triplicate experiments measuring DDX3 level in more than 100 cells. ****$P \leq 0.0001$ (Student's *t*-test).

Supplementary data 3. (A) Huh-7 were treated with either Sor, Pat, or both as described in figure 4. Cells were then collected and their proteins extracts processed for western blot analysis using specific antibodies to the indicated proteins. (B) Huh-7 were treated with Sor, Sor+Tor, Tor, Sor+pp242, or pp242. Cells were lysed and their protein contents analyzed for the expression of ATF4. Tub serves as a loading control. Hypophosphorylation of 4E-BP1 (lower band) serves as a control for mTORC1 inactivation. Quantifications of the results shown at the right are the mean of three independent experiments. (C) Huh-7 were treated with Thap, Thap+Tor, Tor, Thap+pp242, or pp242. Cells were then collected and their protein contents analyzed for the expression of the indicated proteins as above.

Supplementary data 4. The association of DDX3 with the eIF4F complex which occurs during Thap treatment is disrupted by mTORC1 inhibitors. Hep3B were treated with Thap, pp242 or both. (A) Cells were harvested and their lysates were quantified. Equal amounts of lysates were proceeded for cap-binding assays as described in figure 4. Bound eIF4F complexes were eluted and analyzed by Western blot using specific antibodies. (B) DDX3 and eIF4GI enrichments in the m^7GTP binding eluates recovered from Thap-, pp242-, and Thap+pp242-treated cells were estimated as described in figure 4.

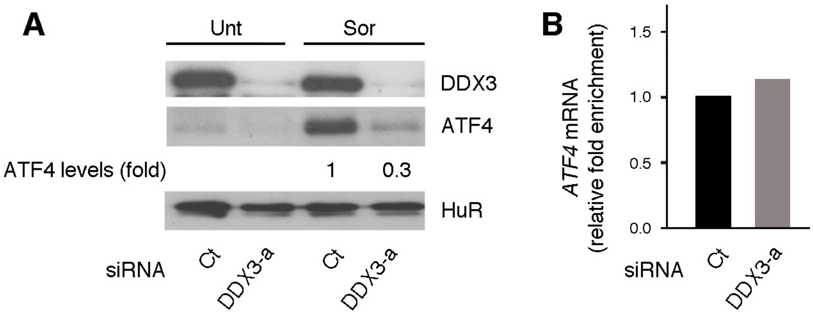
A
Unt
Sor
DDX3
ATF4
ATF4 levels (fold)
1
0.3
HuR
siRNA
Ct
DDX3-a
Ct
DDX3-a
B
ATF4 mRNA
(relative fold enrichment)
1.5
1.0
0.5
0.0
siRNA
Ct
DDX3-a

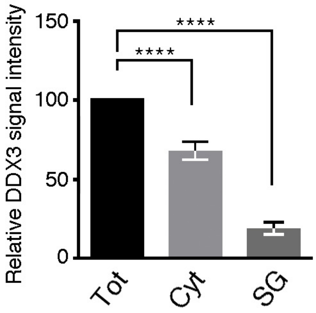

Relative DDX3 signal intensity
150
100
50
0
****
****
Tot
Cyt
SG

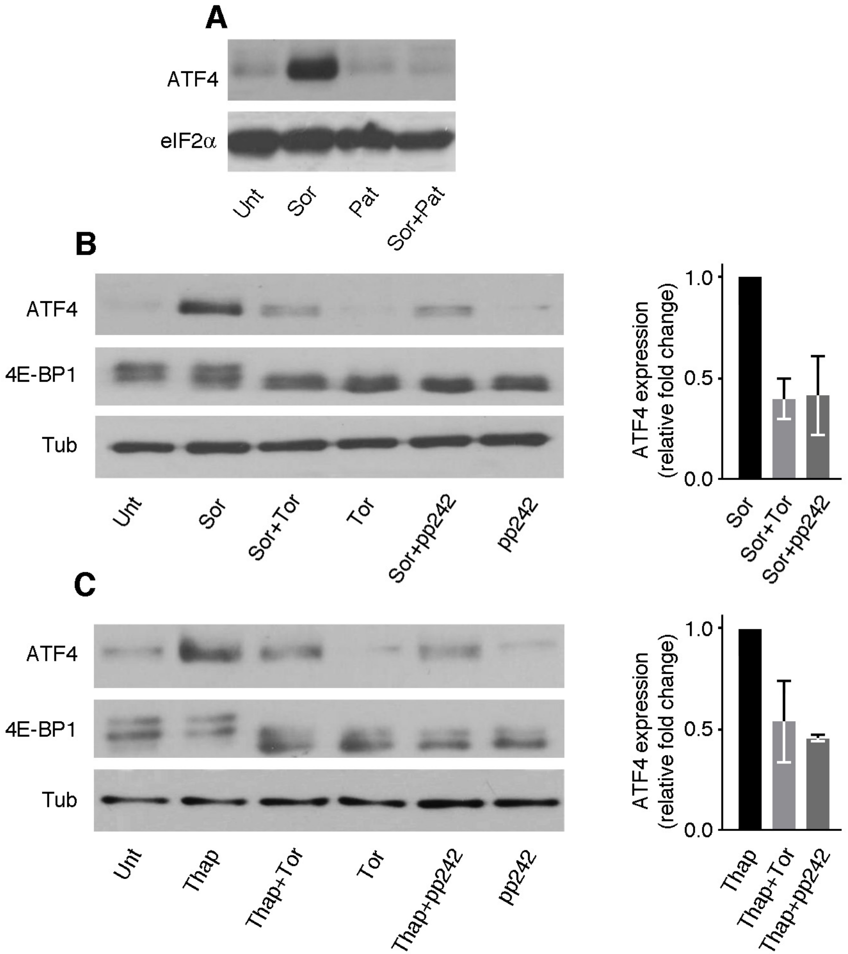
A
ATF4
eIF2α
Unt
Sor
Pat
Sor+Pat
B
ATF4
4E-BP1
Tub
Unt
Sor
Sor+Tor
Tor
Sor+pp242
pp242
ATF4 expression
(relative fold change)
1.0
0.5
0.0
Sor
Sor+Tor
Sor+pp242
C
ATF4
4E-BP1
Tub
Unt
Thap
Thap+Tor
Tor
Thap+pp242
pp242
ATF4 expression
(relative fold change)
1.0
0.5
0.0
Thap
Thap+Tor
Thap+pp242

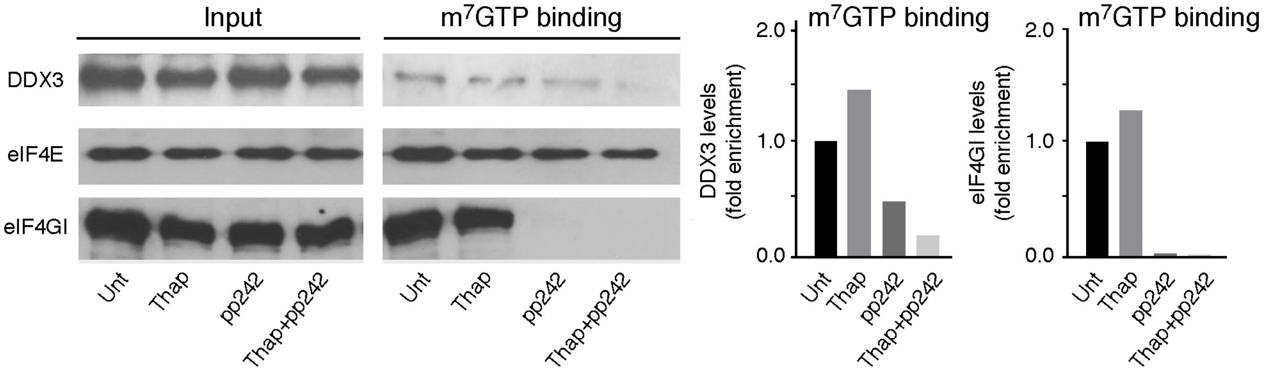
Input
m7GTP binding
DDX3
eIF4E
eIF4GI
Unt
Thap
pp242
Thap+pp242
m7GTP binding
DDX3 levels (fold enrichment)
2.0
1.0
0.0
m7GTP binding
eIF4GI levels (fold enrichment)
